# Vertical Head Movements Influence Memory Performance for Words With Emotional Content

**DOI:** 10.3389/fpsyg.2019.00672

**Published:** 2019-03-26

**Authors:** Laura K. Globig, Matthias Hartmann, Corinna S. Martarelli

**Affiliations:** ^1^Department of Psychology, University of Bern, Bern, Switzerland; ^2^Faculty of Psychology, Swiss Distance Learning University, Brig, Switzerland

**Keywords:** valence, memory, head movements, embodied cognition, space

## Abstract

Numerous studies have found an association between valence and the vertical dimension of space (good-up, bad-down). This association has also been linked to sensorimotor experiences (e.g., body movements). In this study, we investigated whether body movements along the vertical plane play an active role in the retrieval of positive and negative words (as well as words with a more explicit association with up and down). Twenty-five participants were presented with a list of nouns associated with space (e.g., satellite, underground) and a list of nouns associated with emotions (e.g., joy, war). Subsequently, they had to retrieve the words while performing vertical head movements. We found a vertical effect in that participants retrieved more positive words when moving their head upward and more negative words when moving the head downward. These results illustrate that overt body movements are indeed associated with emotional information and can thereby influence what we remember. We conclude that abstract concepts such as emotional representations are inherently linked to motor action and are grounded in space.

## Introduction

Lakoff and Johnson’s (1980) theory of metaphorical representation highlights that aspects of the concrete domain, such as space or bodily states, are used to understand the abstract domain. Motor activities play a functional role in cognitive processes (cf. Niedenthal et al., 2005) and cognition is shaped by an individual’s sensorimotor experience and interactions with the environment (Glenberg et al., 2013). Embodiment theories focus on mental metaphors which allow the mapping of abstract concepts using concrete structures – so-called source domains (cf. Lakoff and Johnson, 1980; Boroditsky, 2000; Casasanto, 2009; Dudschig et al., 2015; Winter et al., 2015).

The *good is up* metaphor postulates a relationship between vertical spatial position and valence (Chasteen et al., 2010; Larson et al., 2012; Sasaki et al., 2016; Woodin and Winter, 2018) and vertical body movements interact with emotional processing (Dudschig et al., 2015). The concept of polarity correspondence assumes that polarity congruent items (positive-up, negative-down) should be categorized faster (Lakens, 2012). Further, positive stimuli trigger or facilitate upward body movements (jumping for joy) and negative emotions lead to downward body movements (slumping shoulders) (Oosterwijk et al., 2009; Dudschig et al., 2015). Gozli et al. (2013) explored metaphorical congruency effects by coupling a conceptual evaluation task with a visuospatial task. Saccade trajectories exposed a tendential vertical deviation pattern driven by positive concepts.

Emotions are not only expressed through body movements, but body movements also modulate the emotions we experience. Participants recalled negative autobiographical memories faster when performing downward arm-movements. They also reported more negatively valenced memories. The reverse was true when upward arm-movements were performed (Casasanto and Dijkstra, 2010). This indicates a bi-directional link between emotional valence and space. The relationship between motion and emotion was further assessed using a passive motion task (Lachmair et al., 2016). Recall was facilitated when body position of participants (upright or head-down tilted) was congruent with spatial words. Recently, Casasanto and de Bruin (2019) extended prior findings to show that motor action may also serve to enhance learning of simple words.

This study aims to further explore the *good is up* metaphor and determine its role during recall of words associated with emotion. We investigated the relationship between motor action along the vertical axis and recall of words associated with emotion. Target stimuli associated with space were used to allow for comparison between the effect of overt motor action on recall of words pertaining to a concrete domain (space) and an abstract domain (emotion).

In our study, participants encoded positive, negative, up, down, or neutral items. During recall, participants alternatingly moved their head up and down. Vertical head movements interfere with the activation of up- and down-associated concepts such as number magnitudes (cf. Loetscher et al., 2008; Winter and Matlock, 2013)^1^. We hypothesized that participants retrieve more positive words when moving their head upward and more negative words when moving their head downward. Confirmatory findings would add evidence to the association between positively and negatively valenced words and vertical motions (Meier and Robinson, 2004). Secondly, we hypothesized that participants recall more up-words when moving their head upward and more down-words when moving their head downward. Location is activated when nouns associated with up- or down-locations are processed (Lachmair et al., 2011).

## Materials and Methods

### Participants

An *a priori* G^∗^Power analysis revealed that we needed a sample of 28 participants to detect at least a medium effect (2 × 3 analysis of variance with repeated measures in both factors; parameters: *f* = 0.25, α = 0.05, 1-β = 0.80, ρ = 0.5; Faul et al., 2007). We collected data from 28 participants recruited from the student population of the University of Bern. Participants were naïve about the experiment’s purpose and gave written-informed consent. We included 25 participants (52% women, mean age = 25.4, *SD* = 4.52) in analyses. Two participants were excluded due to discovering the hypothesis during their experimental session. Another participant was excluded due to very low memory performance in the task with valenced words (4/45 words recalled). The local Ethics Committee approved the study, which was conducted according to the principles of the Declaration of Helsinki.

### Materials and Design

In our task, participants encoded and recalled items. Participants were presented with two counterbalanced blocks of word lists, one associated with space and another associated with emotion. The list of 45 words associated with space was taken from Lachmair et al. (2011). It consists of nouns referring to entities associated with either up- (e.g., satellite), center- (e.g., train) or down-locations (e.g., underground). The list of 45 words associated with emotion was taken from the Berlin Affective Word List Revised (BAWL-R) by Võ et al. (2009). The nouns refer to entities associated with positive (e.g., joy), neutral (e.g., auditory) or negative valence (e.g., war). To prevent hypothesis discovery and to allow for isolation of a possible main effect of head movement on recall neutral items were included. Based on the BAWL-R the three categories differed in emotional valence, *p* < 0.001 (positive: *M* = 2.60, *SD* = 0.14; neutral: *M* = 0.10, *SD* = 0.40; negative: *M* = -2.74, *SD* = 0.07). Further, the word lists related to emotion did not differ significantly in word length between positively valenced (*M* = 6.53, *SD* = 1.13) and negatively valenced words (*M* = 6.53, *SD* = 1.96) [*t*(28) = 0, *p* = 1]. There was also no significant difference in word length between up (*M* = 6.13, *SD* = 1.96) and down words (*M* = 5.80, *SD* = 1.74) [*t*(28) = 0.49, *p* = 0.63]. With regards to word frequency, research found that everyday language use is positively biased so that positive words are more likely to be used compared with negative words (cf. Garcia et al., 2012). Further, the DWSD database (Klein and Geyken, 2010), allowed us to control for relative frequency of word usage in text and media corpora in both emotionally valenced and spatial words. Indeed with regards to the frequency information provided we found that positive (*M* = 4.67, *SD* = 0.49) and negative words (*M* = 4.27, *SD* = 0.46) differed significantly [*t*(28) = 2.32, *p* = 0.03]. With regards to spatial words we found that up (*M* = 4, *SD* = 0.85) and down (*M* = 3.73, *SD* = 0.76) words did not differ significantly [*t*(28) = 1.07, *p* = 0.29]. (All words are reported in the section “Appendix”).

### Procedure

Participants were asked to learn the first list of words (counterbalanced, either associated with space, or emotion). Words were presented on a computer screen in random order for 5 s per word using Psychopy. Participants then executed a random number generation task while performing vertical head movements (40 up-down head movements, participants generated random numbers between 1 and 30). This task acted as a distraction task and entrained the head movements. During this task the experimenter intervened if participants executed movements incorrectly.

Finally, participants had to retrieve (free recall) the words while performing the previously learned head movements. The experimenter did not intervene during the mental recall stage. Participants were instructed to move their heads continuously to the pace of a metronome (3 s). They were filmed to ensure proper task execution. To prevent hypothesis discovery a cover story (see section “Appendix”) was used. This informed participants that the study sought to assess the effect of bodily motions on memory performance in a movement and a no-movement group. In fact, the study did not include a no-movement condition. Finally, participants were asked what they conceived the study was about.

## Results

The analyses focus on the mean number of words recalled. Based on previous findings (Casasanto and Dijkstra, 2010) we expected better performance when motion and valence or motion and space, respectively, were congruent than when they were incongruent. We computed repeated measures analyses of variance (ANOVAs) and planned contrasts between upward and downward head movements and report partial eta squared ($\eta_{p}^{2}$) as measure of effect size. No mathematical correction was made for multiple comparisons. The values of asymmetry and of kurtosis for the different variables were between -1.30 and 0.93, thus considered acceptable in order to prove normal univariate distribution. Sphericity was met (Mauchly’s test reached *p*-values > 0.05). These analyses were computed with IBM SPSS version 25. To quantify how much the data should shift our belief in favor of the null or the alternative hypothesis, we computed Bayes Factors (BF_10_ where 1 means that the two hypotheses are equally likely, larger values indicate more evidence for the alternative hypothesis, and smaller values indicate more evidence for the null hypothesis). All Bayesian analyses were computed with JASP version 0.9.1. Given the small sample, we additionally report non-parametric tests, which were computed with JAMOVI version 0.9.5.5.

### Words Associated With Emotion

**FIGURE 1:**
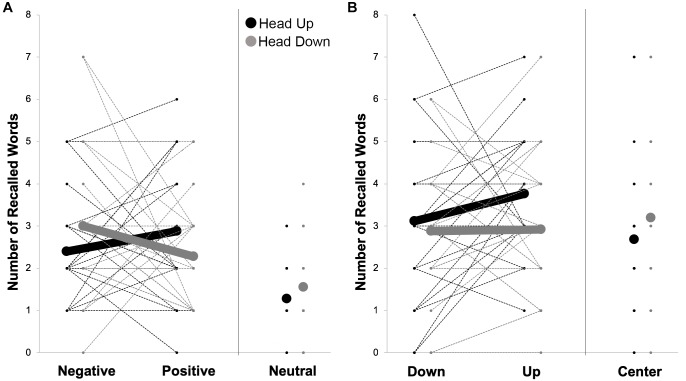
Number of recalled words for items associated with emotion (negative, positive, and neutral – **A**) and for items associated with space (down, up, and center – **B**) separated by up and down head turns. Means (in bold) and individual scores are reported.

On average, participants recalled 13.40 words out of 45 (*SD* = 5.36). We conducted a repeated measures ANOVA with word type (negative, neutral, positive) and head movements (downward, upward) as within-subjects factors and the mean number of recalled words as dependent variable. The main effect of word type was significant, *F*(2,48) = 14.05, *p* < 0.001, $\eta_{p}^{2}$ = 0.37, BF_10_ = 356’982.62. On average participants remembered more negative (*M* = 2.70, *SEM* = 0.27) and more positive words (*M* = 2.58, *SEM* = 0.23) compared to neutral words (*M* = 1.42, *SEM* = 0.20). The main effect of head movements was not significant, *F* < 1, BF_10_ = 0.000001286. The analysis revealed a significant interaction, *F*(2,48) = 4.66, *p* = 0.014, $\eta_{p}^{2}$ = 0.16, BF_10_ = 166’555.94. Planned comparisons (simple effects of head movements within level of type of word) showed a significant difference between upward and downward head movements within negative items: during upward head movements participants recalled on average 2.40 (*SEM* = 0.25) negative words and during downward head movements participants recalled on average 3.00 (*SEM* = 0.34) negative words, *F*(1,24) = 6.35, *p* = 0.019, $\eta_{p}^{2}$ = 0.21, *Wilcoxon W* = 163.00, *p* = 0.025, BF_10_ = 2.83. The head movement manipulation had no influence on positive words, *F*(1,24) = 3.27, *p* = 0.083, $\eta_{p}^{2}$ = 0.12, *Wilcoxon W* = 52.50, *p* = 0.083 BF_10_ = 0.86. See Figure 1.

### Words Associated With Space

On average participants remembered 18.56 out of 45 words (*SD* = 7.90). We conducted a repeated measures ANOVA with word type (down, center, up) and head movements (down, up) as within-subjects factors and number of recalled words (mean) as dependent variable. This analysis revealed no significant result (*ps* > 0.103, BFs_10_ < 0.244). Even though the interaction was not significant, *F*(2,48) = 5.81, *p* = 0.103, $\eta_{p}^{2}$ = 0.09, BF_10_ = 0.074, planned comparisons (simple effects of head movements within level of word type) showed a significant difference between up and down head movements within up items: during upward head movements participants recalled on average 3.76 (*SEM* = 0.29) up-words and during downward head movements participants recalled 2.92 (*SEM* = 0.34) up-words, *F*(1,24) = 5.62, *p* = 0.026, $\eta_{p}^{2}$ = 0.19, *Wilcoxon W* = 38.50, *p* = 0.022, BF_10_ = 2.155. No differences between upward and downward head movement conditions within down-words were found, *F*(1,24) = 0.37, *p* = 0.547, $\eta_{p}^{2}$ = 0.015, *Wilcoxon W* = 101.50, *p* = 0.634, BF_10_ = 0.250. See Figure 1.

## Discussion

We sought to investigate the effect of overt head movements on memory performance during recall of items associated with space (down, center, and up) and emotion (negative, neutral, and positive). We show that the concrete domain of motion and the more abstract domain of emotion are indeed functionally related. This is consistent with the *good is up* metaphor.

In the abstract domain of emotion, we found a vertical effect of head movements on recall: Participants retrieved more negative words when moving their head downward. Thus, body movements are likely to be associated with emotional information and may interact with the recall of valenced memory contents. This supports prior findings that retrieval is facilitated when motion and memory valence are congruent (Casasanto and Dijkstra, 2010). We also extend these findings. Not only did we use a different motor task (vertical head movements vs. moving marbles) but we also used controlled stimulus material instead of subjective autobiographical memories. Using simple nouns allowed us to control for emotional valence to draw more general conclusions. Body movements – specifically overt head movements – can influence our ability to recall emotionally valenced material.

Planned comparisons showed that participants retrieved more up-words when moving their head upward. This is consistent with previous literature emphasizing the close relationship between the two concrete domains of space and motion (Lachmair et al., 2011). However, head movement manipulation affected emotional memories more than spatial memories. This is surprising as studies show that location is automatically activated when nouns associated with up- or down-locations are processed (e.g., Lachmair et al., 2011). Lachmair et al. (2016) found a stronger relationship between motion and space than between motion and valence. Contrastingly, we found indications for an effect of motion on valence but less for an effect of motion on space. What could account for this different pattern of results?

The effect of vertical head movements on retrieval may be moderated by the level of difficulty. Free recall of words associated with space was slightly easier than free recall of words associated with emotion. On average, participants remembered 19 words associated with space and 13 words associated with emotions. A possible limitation of our study might be that the task was rather difficult, resulting in relatively low rates of recall. Future research should compare a variety of levels of difficulty with regards to recall. Further, one must note the difference in tasks. Whereas in our study participants conducted head movements only during recall, motion took place during encoding in Lachmair et al. (2016). Moreover, manipulation of body posture in previous studies was usually static. We relied on continuously conducted movements. Effects in previous studies centered around response times. Faster response times are indicative of automatic activation and facilitatory effects. Instead, our study tested retrieval. A limitation of our study as a conceptual replication of previous evidence as such is the absence of a comparison of how different tasks and manipulation during either encoding or retrieval affect memory performance. Further we did not include a neutral head position condition in which participants only face straight-ahead. Future studies should address these limitations to explore the relevance of employed task manipulation and the proposed concept of functionality further. Additionally, further studies, including a larger sample size should re-examine the effect for space-related words. We sought to determine the link between motion and emotion with a controlled recall task. Results revealed a direction-specific influence of head movements on retrieval of words with emotional content. We conclude that abstract concepts such as emotional representations are inherently linked to motor action and grounded in space.

## Data Availability

Dataset available at https://figshare.com/s/e8067a5eac402954c657.

## Author Contributions

MH and CM conceived the study. CM performed the formal analysis. MH visualized the study. LG and CM wrote the manuscript. LG, MH, and CM reviewed and edited the manuscript.

## Conflict of Interest Statement

The authors declare that the research was conducted in the absence of any commercial or financial relationships that could be construed as a potential conflict of interest.

## Footnotes

1 We chose to manipulate head movements as numerous studies have associated head movements as characteristic of valence expressions, including pride and shame (cf. Tracy and Matsumoto, 2008). They thus have an inherently integral role in our day to day lives, while being simple enough to isolate.

## Appendix 1

**Table 1:** List of stimuli used during the task (German and English translation). The words associated with space were taken from Lachmair et al. (2011). The words associated with emotion were taken from the Berlin Affective Word List Revised (BAWL-R) by Võ et al. (2009).

Up	Down	Center	Positive	Negative	Neutral
Weltall	Untergrund	Zug	Musik	Weltkrieg	Ablauf
Höhe	Tunnel	Lastwagen	Harmonie	Bluttat	Absender
Vogelnest	Tümpel	Torte	Idylle	Tyrann	Bericht
Ballon	Graben	Locher	Sommer	Alptraum	Achse
Satellit	Keller	Brief	Wahrheit	Qual	Akzent
Dach	Grab	Maschine	Zuhause	Folter	Aussage
Vogel	Gruft	Kaffee	Frieden	Massaker	Bewerber
Zepellin	Tiefe	Zigarre	Freund	Mord	Lesesaal
Hochsitz	Klee	Bär	Ferien	Pest	Geruch
Krone	Schienen	Kleidung	Heilung	Atombombe	Hausfrau
Sonne	Wurzel	Neuwagen	Glück	Tumor	Hypnose
Ufo	Schlucht	Hecke	Freude	Gewalt	Inhalt
Hochland	Sohle	Bier	Freiheit	Faschismus	Karton
Berg	Maus	Buch	Paradies	Untreue	Kollektiv
Hochhaus	Gras	Metropole	Liebe	Angst	Lösung
Space	Underground	Train	Music	World War	Process
Altitude	Tunnel	Lorry	Harmony	Blood Crime	Sender
Bird’s nest	Pond	Torte	Idyll	Tyrant	Report
Balloon	Trench	Hole Punch	Summer	Nightmare	Axis
Satellite	Cellar	Letter	Truth	Torment	Accent
Roof	Tomb	Machine	Home	Torture	Statement
Bird	Crypt	Coffee	Peace	Massacre	Applicant
Zeppelin	Depth	Cigar	Friend	Murder	Reading room
Deer Stand	Clover	Bear	Holiday	Pest	Scent
Crown	Rails	Clothes	Healing	Atomic Bomb	Housewife
Sun	Root	New Car	Fortune	Tumor	Hypnosis
UFO	Canyon	Hedge	Joy	Violence	Content
Highlands	Sole	Beer	Freedom	Fascism	Cardboard
Mountain	Mouse	Book	Paradise	Infidelity	Collective
Skyscraper	Grass	Metropolis	Love	Fear	Solution

## Cover Story

*This experiment investigates the influence of head movement on memory. There is one group with and one group without head movement. You are in the group with head movement. All participants in your group, perform various head movements*.

NB. The experiment did not include a no-movement condition. This information was given to make the cover story believable.
